# Comparing assessment of diabetes-related quality of life between patients and their physicians

**DOI:** 10.1186/s12955-018-1040-6

**Published:** 2018-11-19

**Authors:** Orly Tamir, Nitzan Shabo De-Paz, David Dvir, Anthony D. Heymann

**Affiliations:** 1 The Gertner Institute for Epidemiology and Health Policy Research, Sheba Medical Center, Ramat Gan, Israel; 2 D-Cure Foundation, Petach-Tikva, Israel; 3 Sackler Faculty of Medicine, Tel Aviv University, Tel Aviv, Israel; 4 Maccabi Healthcare Services, Tel Aviv, Israel

**Keywords:** Type 2 diabetes, Patient perception, Concordance, Patient-centered care, Quality of life (QoL)

## Abstract

**Background:**

Health-related quality of life (QoL) is a comprehensive, multidimensional construct encompassing physical and psychosocial wellbeing. Physicians frequently assess QoL as part of their decision making process without specifically asking their patients. This study examined the degree and predictors of concordance between physician and patient assessments of QoL among patients with diabetes in primary care and in multi-disciplinary diabetes clinics.

**Methods:**

Patients completed a questionnaire regarding overall and diabetes-specific QoL before entering their physician’s office. After the visit, the physician completed the same questionnaire in order to evaluate how he/she perceived that patient’s QoL. In addition, medical data relating to the patient’s health status were collected from the medical records. The concordance between patient-reported QoL and physician-estimated QoL was evaluated. Stepwise regression analysis was conducted to determine which factors contributed to the difference between physicians’ and patients’ assessment of QoL.

**Results:**

A total of 136 patients and 39 treating physicians were surveyed. Patients’ response rate was 95%. A strong concordance was found between patients’ and physicians’ ratings of current health status (*r* = 0.79, *p* < 0. 01); however, physicians perceived their patients’ QoL as worse than the QoL assessed by the patients themselves. Primary care physicians were better at assessing their patients’ overall wellbeing while diabetes-specialists were better at assessing their patients’ diabetes-specific QoL. In addition, the longer the duration of diabetes, the more difficult is was for the physicians to accurately assess QoL. When entered in the regression analysis, familiarity did not explain physicians’ ability to assess health-related QoL or diabetes-specific QoL.

**Conclusions:**

Physicians make reasonable assessments of their patients’ QoL, however as the patients’ disease progresses, it becomes harder for physicians to assess QoL. Primary care physicians are better at assessing overall well-being whereas diabetes specialists are better at assessing diabetes-specific QoL.

**Trial registration number:**

Not registered. Assuta Medical Center institutional review board approval number 2009103.

## Background

Health-related quality of life (QoL) is a comprehensive, multidimensional construct encompassing physical and psychosocial wellbeing [1]. The importance of incorporating QoL considerations in the choice of treatment strategy has long been acknowledged in various health conditions [2, 3]. However, outside the realm of research, assessment of patients’ health-related QoL is unlikely to happen because of the time constraints in everyday practice. It is thus important to understand whether physicians can assess their patients’ health-related QoL in a manner that may be helpful for treatment decisions.

Physicians frequently make proxy judgments of QoL as part of their decision making process without specifically asking their patients about it. Since these proxy judgements can influence the choice of treatment, it is important to know if they are in concordance with the patients’ assessments. This is especially true for the management of chronic diseases such as diabetes, whereby self-management of care can be a real burden for patients with this condition. In diabetes, improved health-related QoL is a therapeutic target in and of itself, and some studies suggest that it can also promote optimal metabolic outcomes [4]. In diabetes care, the range of treatment options is large and certain treatments, such as those involving injections, can worsen the health-related QoL. Therefore, it is important that the physicians’ judgement is accurate.

The use of proxy rating in health-related QoL assessment is much debated in the medical literature. A large body of research has been dedicated to studying the possibility of QoL assessment by proxy in populations that are limited in their ability to provide accurate answers to QoL questions, such as children, the critically-ill, people with severe mental illness and patients suffering from dementia. To that end, various studies have compared responses of patients to those of their direct care-givers, such as family members or long-term care-takers, as well as to those of medical personnel, including physicians and nurses. However, the degree of congruence between the self-assessments of patients’ health-related QoL and assessments made by primary care-takers, mostly family members, varies widely between studies, and the directionality of this difference is not uniform [5–8].

A few studies that evaluated directly the agreement between the self-assessment of health-related QoL and health practitioner assessments have been published in the past decade. Rafique and Naqvi showed that the correlation of New-York Heart Association class with QoL score was significantly better for patients’ self-assessment compared to the assessments performed by cardiologists [9]. Costello et al. reported that clinicians perform poorly when asked to predict Health-related QoL for children with cardiac disease: they tended to overestimate Health-related QoL for those patients and parent-proxies who reported lower Health-related QoL, and to underestimate Health-related QoL for those reporting higher Health-related QoL [10]. In a cross-sectional study of 270 female breast cancer patients, the patient-oncologist agreement was weak to moderate as patients tended to rate themselves worse than the oncologists’ assessment [11]. A different study in 148 patients with breast cancer showed mostly moderate to good agreements between QoL scores (assessed using the European Organization for Research and Treatment of Cancer Quality of Life Questionnaire) of patients and those of their oncologists [12]. In a study that compared neurologist and patient perceptions of multiple sclerosis-related health status, significant differences were noted between patient and neurologist ratings for QoL. Neurologists identified physical functioning domains as important, while patients placed more emphasis on mental health domains [13].

In this study, our aim was to assess the degree and predictors of concordance in health-related QoL assessment between adult patients with diabetes and that of their diabetes-treating physicians. The assessed predictors were sociodemographic and disease related. Further, we attempted to evaluate whether the ability to serve as adequate proxies for the patients in this regards differs between family physicians and diabetes specialists.

## Methods

### Study population and setting

This cross-sectional study was conducted between August 2011 and December 2012 in 14 regional and district clinics across three administrative and geographical regions of Israel (Sharon, Central, and the Jerusalem & Coastal plain). The clinics comprised 9 primary care clinics (PCC) and 5 multi-disciplinary diabetes-specialty clinics (MDC), that are part of Maccabi Healthcare Services the second largest health maintenance organization in Israel insuring two million members countrywide, 25% of the total Israeli population. According to the Israeli National Health Insurance Act, Maccabi Healthcare Services must accept all applicants, and therefore every sector in the Israeli population is represented. Nonetheless, coverage in the non-Jewish populations is substantially lower compared to the Jewish population.

Data on all members’ interactions including hospitalizations, outpatient visits, laboratory test results and dispensed prescriptions by Maccabi Healthcare Services pharmacies is downloaded daily to a central computerized database. All patients purchase their medication at Maccabi Healthcare Services pharmacies so each patient has a record of medication purchase and concordance with prescribed treatment.

### Sampling of the study population

The clinics were chosen according to the cluster sampling method whereby mutually homogeneous yet internally heterogeneous groupings are evident in a statistical population. Subjects were recruited to the study by convenience sampling. This non-probability sampling method relies on data collection from population members who are conveniently available to participate in study [14]. For each clinic in the study, on days that at least two physicians were scheduled to simultaneously see patients, a researcher was present at the clinic’s waiting areas during opening hours. The researcher recruited patients to participate in the survey during routine clinical visits while waiting to see their treating physicians. All eligible patients were asked for their written informed consent, and were interviewed (face-to-face) immediately by a trained interviewer. Physicians, who had previously given their oral consent to participate in the study, were asked to complete the same questionnaire for each of their patients who completed the questionnaire in order to examine their perception of their patient’s health-related QoL, as well as their familiarity with their patient’s condition and health status. The physicians completed each questionnaire on the same day of the patient’s visit or during the following day. For each physician, no more than 6 patients were recruited for this study.

The calculation of the minimal sample size required in the study was based, in part, on results obtained in previous similar studies that examined the agreement between patients and physicians on quality of life issues and found a statistically significant difference [7, 15, 16]. In the current study, an additional calculation was made for the minimum sample size required in each organizational framework using the WINPEPI33 software. The Inference for Means - Comparing Two Independent Samples test was calculated based on the following assumptions: 80% test strength, 5% significance level, 30% mismatch between patients and physicians, and 15% difference between group averages. Assuming there will be 6 patients to each physician [17], the minimum required sample size was 126 patients (63 in each type of clinic) and 22 physicians (11 in each type of clinic). The study actually included 136 patients with diabetes (70 were interviewed at primary care clinics and 66 were interviewed at multi-professional diabetes clinics), and 39 physicians (28 family physicians and 11 diabetes specialists); therefore, assuming that the sample size is 136 and the proportion in the sample is 30% at a confidence level of 95%, the difference between the proportion obtained in the sample and the true proportion does not exceed 7.7% (±). Therefore, it is possible to determine with 95% confidence that the true proportion ranges from 22.3 to 37.7%.

### Data collection

The written answers to the structured questionnaire were answered by the patient while in the waiting area before his appointment. The questionnaire comprised of the following inventories:

- **The European Quality of Life 5-Dimensions 3 levels (EQ-5D-3 L) questionnaire** - The EQ-5D questionnaire comprises two components: a health state description and an evaluation. In the description section, five dimensions are evaluated: mobility, self-care, usual activities, pain/discomfort, and anxiety/depression. The respondents self-rate their level of severity for each dimension using a three-level scale, whereby 1 represents no deficiency and 3 represents complete deficiency.
  In the evaluation part of the EQ-5D-3 L, the respondents indicate their current perceived overall health status using a visual analogue scale (EQ-VAS) that ranges from zero (“worst imaginable health state”) to 100 (“best imaginable health state”) indicating the patient’s current perception of his health state [18].
  In addition to the two measures described above, the patient and physician ratings combinations of the health state description were transformed into six profile groups (A-F) that provide a general assessment of the patients’ QoL. The “A Profile” indicates the best health while the rest of the profile groups indicated a sub-optimal rating in one or more health dimensions, with the “F Profile” representing the worst health.
- **Diabetes QoL Brief Clinical Inventory questionnaire (DQOL–BCI) –** is 15-item diabetes-specific tool that provides one total score ranging from 1 to 5 (lower score is better QoL) that predicts self-reported diabetes care behaviors and satisfaction with control.
- **The patient-perceived difficulty in diabetes treatment (PDDT) scale** - contains 12 items reflecting diabetes-treatment characteristics: adherence to self-monitoring of glucose schedule, frequency of self-monitoring of glucose, adherence to medication administration schedule, frequency of medication administration, multiple number of medications, synchronization between meals and medications, dependence on the medications, pain associated with treatment, diet restrictions, self-care, multiple healthcare providers, and costs of treatment. The difficulty of each characteristic rated on a scale ranging from 1 (=very difficult) to 5 (=not difficult at all) [19, 20].
  Full description of translation and validation of all of the questionnaires in Hebrew has been previously reported [19–21].
  In addition to the above-mentioned questionnaires, the patients rated their satisfaction concerning their relationship with the clinic’s medical personnel (on a scale ranging from 1 = ‘not at all’ to 5 = ‘very much’), and their perception of the extent of physicians’ familiarity with them (on a scale ranging from 1 = ‘not at all’ to 10 = ‘very much’). The patients were also asked to note socio-demographic (i.e. age, gender and education) and diabetes-related (i.e. disease duration, type of medication therapy, comorbidities and complications) variables.
  Data were also extracted from the patients’ computerized medical records. These data included purchase of diabetes medication which we used as a proxy for adherence and recent values of clinical measures including: glycated hemoglobin A1c (HbA1c), systolic and diastolic blood pressure (SBP/DBP), body mass index (BMI), triglycerides (TG), low-density lipoprotein (LDL)-cholesterol and urine albumin to creatinine ratio (ACR).

### Outcome measures

The primary outcome measure of the study was the degree of concordance in health-related QoL assessment between adult patients with diabetes and that of their diabetes-treating physicians.

Additional outcome measures included predictors of agreement of health-related QoL assessments between the patients and their physicians, and the ability of physicians and diabetes specialists to serve as adequate proxies for their patients.

### Data analysis

Data were analyzed using SAS version 9.1 (SAS Institute Inc., Cary, NC, USA). Categorical variables were described as numbers and percentages and continuous variables were expressed as mean ± standard deviation (SD). Bivariate analysis was conducted using chi-square or Fisher’s exact tests for categorical variables and the independent sample t-test for continuous variables. Pearson’s correlation coefficient was used to determine the direction and degree of association between continuous study variables. Agreement between patient and physician health-related QoL scores in the descriptive system was determined by calculating weighted kappa (K) scores and agreement percentage**.** Differences between patient and physician health-related QoL scores were analyzed with the paired sample t-test, and the independent sample t-test was used to determine statistically significant differences in mean score between groups in PCCs and MDCs. Multiple linear regression analysis was conducted using two separate stepwise-selection approach regression models to determine which factors contributed the most to the prediction of the mean relative score difference of the patient’s current health status (EQ-VAS continuous variable) and the diabetes-specific QoL (DQOL–BCI continuous variable) as the dependent variables. Variables included in the regression models were those that had significant association levels with the dependent variable in the univariate analysis (*p* < 0.01). An alpha level of 0.15 was the default used for the stepwise selection approach. In addition, interactions between the independent variables included in the model were tested within the regression model. All statistical comparisons were two-sided and significance was defined as *p* < 0.01.

## Results

### Study population characteristics

A total of 136 patients with type 2 diabetes mellitus (70 from PCCs and 66 from MDCs) and 39 treating physicians (28 family physicians and 11 diabetes specialists) were surveyed, with an overall patient response rate of 95%. There were no significant differences between respondents and non-respondents in age, gender or type of care-providing clinic.

Compared to patients treated in PCCs, patients treated in MDCs had significantly higher levels of LDL cholesterol (*p* < 0.01), systolic blood pressure (*p* < 0.01), urine albumin to creatinine ratio (*p* < 0.01), as well as longer disease duration (*p* < 0.01), a higher number of comorbidities (*P* < 0.01) and diabetes-related complications (P < 0.01). Socio-demographic, clinical characteristics, and diabetes-related measures of the study population are detailed in Table 1.

**Table 1:** Selected socio-demographic, clinical characteristics, and diabetes-related measures of study population

	Total population *N* = 136	Diabetes clinic (MDC) *N* = 66	Primary care clinic (PCC) *N* = 70	*P* value
Age (years)				NS
18 ≤ Age < 49 N(%)	14 (10.3)	4 (6.1)	10 (14.3)	
50 ≤ Age < 69 N(%)	80 (58.8)	39 (59.1)	41 (58.6)	
70 ≤ Age N(%)	42 (30.9)	23 (34.9)	19 (27.1)	
Gender: Male N(%)	73 (53.7)	34 (51.5)	39 (55.7)	NS
Years of education^a^				NS
≤12 years N(%)	79 (58.1)	36 (54.5)	43 (61.4)	
> 12 years N(%)	57 (41.9)	30 (45.5)	27 (38.6)	
Body Mass Index (kg/m^2^) Mean (SD)	29.1 (3.3)	28.2 (2.7)	29.9 (3.7)	< 0.01
Diabetes duration (years) Mean (SD)	12.8 (9.4)	15.1 (10.2)	10.6 (8.2)	< 0.01
Number of chronic co-morbidities Mean (SD)	1.7 (1.2)	2.1 (1.1)	1.3 (1.2)	< 0.01
Number of diabetes-related complications Mean (SD)	2.8 (2.7)	4.0 (2.9)	1.7 (2.0)	< 0.01
HbA1c (%)Mean (SD)	7.2 (1.3)	7.4 (1.3)	7 (1.3)	NS
LDL-cholesterol (mg/dl) Mean (SD)	123.5 (53.9)	138.1 (62.1)	109.7 (40.7)	< 0.01
Systolic Blood pressure (mm Hg)^b^ Mean (SD)	132.3 (12.3)	135.6 (11.5)	129.2 (12.4)	< 0.01
Albumin/creatinine ratio (mg/gr)^b^ Mean (SD)	14.2 (34.5)	22.4 (44.4)	6.5 (18.3)	< 0.01
Medication therapy for diabetes				
1 or more oral hypoglycemic agents N(%)	99 (73)	35 (53)	64 (91.4)	< 0.01
Oral hypoglycemic agent + Insulin N(%)	37 (27)	31 (47)	6 (8.6)	< 0.01
Optimal purchase of diabetes medications^b^ N(%)	104 (76.5)	47 (71.2)	57 (81.4)	NS

Categorical variables are presented as n (%) and continuous variables are presented as mean (SD)

*NS* not significant, *SD* standard deviation

^a^Twelve years of education = completed secondary school

^b^Percentage of patients who purchased at least 80% of prescribed monthly prescriptions in previous year

### Evaluation of patients’ health-related QoL by patients and physicians

In the PCC setting, both patients and physicians assessed the patients’ health-related QoL (as measured by the EQ-5D-3 L questionnaire) as better than that of patients in the MDC setting (*p* = 0.04 and *p* < 0.01, respectively; Table 2). In both treatment settings, physicians perceived the patients’ health-related QoL as lower than did the patients themselves, and the difference was larger in MDCs than in PCCs. In all, a high concordance was found between patients’ and physicians’ ratings of ratings of EQ-5D-3 L index score and physicians’ ratings of the same questionnaire (*r* = 0.792, *p* < 0.01).

**Table 2:** Comparison of patient-reported and physician-estimated current health status, QoL, diabetes-specific QoL, and PDDT

	Total population *n* = 136	Diabetes clinic *n* = 66	Primary care clinic *n* = 70	*P* value
Current health status (EQ-VAS)				
Patients’ assessment of current health, Mean (SD)	75.9 (13.5)	71.7 (14.9)	79.9 (10.8)	< 0.01
Physician-estimated patient’s current health Mean (SD)	73.9 (12.5)	68.6 (13.4)	78.7 (9.4)	< 0.01
Concordance between physician-estimated and patient-reported EQ-VAS score, Pearson correlation coefficient (*p* value)	*r* = 0.79 (< 0.01)	*r* = 0.72 (< 0.01)	*r* = 0.83(< 0.01)	
EQ-5D-3 L score^a^				
Patients’ assessment, Mean (SD)	0.75 (0.27)	0.70 (0.294)	0.80 (0.246)	0.04
Physicians’ assessment, Mean (SD)	0.72 (0.293)	0.65 (0.314)	0.79 (0.255)	< 0. 01
Concordance between patient-reported and physician-estimated EQ-5D index score, Pearson correlation coefficient (*p* value)	*r* = 0.79 (< 0.01)	*r* = 0.72, (< 0.01)	*r *= 0.83, (< 0.01)	
Profile A rating^b^				
Patients’ assessment (%)	48.5	43.9	52.9	NS
Physicians’ assessment (%)	44.1	36.4	51.4	0.04
Concordance between physician-estimated and patient-reported profile A rating, weighted Kappa (95% Condidence Interval)	*K* = 0.63 (0.54–0.72)	*K* = 0.61 (0.49–0.73)	*K* = 0.625 (0.48–0.77)	
Diabetes-specific QoL				
Patients’ assessment, Mean (SD)	1.91 (0.62)	2.21 (0.69)	1.72 (0.48)	< 0.01
Physicians’ assessment, Mean (SD)	1.93 (0.72)	2.21 (0.78)	1.67 (0.55)	< 0. 01
Concordance between patient-reported and physician-estimated diabetes-specific QoL score, Pearson correlation coefficient (p value)	*r* = 0.61 *Ρ* = 0. 01	*r* = 0.67 *Ρ* = 0. 01	*r* = 0.35 *Ρ* = 0. 01	
Perception of Difficulty with Diabetes Treatment (PDDT)^c^				
Patients’ assessment, Mean (SD)	4.12 (0.84)	3.74 (0.92)	4.48 (0.55)	< 0.01
Physicians’ assessment, Mean (SD)	4.09 (0.83)	3.71 (0.86)	4.45 (0.62)	< 0.01
Concordance between patient-reported and physician-estimated PDDT score, Pearson correlation coefficient (p value)	*r* = 0.59 (< 0.01)	*r* = 0.49 (< 0.01)	*r* = 0.52 (< 0.001)	

*NS* not significant, *SD* standard deviation

^a^Higher scores reflect better quality of life

^b^Profile A: indicates no problems in any of the 5 dimensions of the EQ-5D

^c^Lower score reflects better quality of life

Physicians in PCCs rated a higher percentage of patients as “Profile A” than physicians in MDCs (51.4% vs. 36.4%, *p* = 0.04). Although the percentage of “Profile A” patients, as rated by the patients themselves, was also higher in PCCs than in MDCs (52.9% vs. 43.9%), the difference was not statistically significant. As presented in Table 2, the concordance between physician- and patient-rated “Profile A” was high in both settings, though slightly higher in PCCs than in MDCs (K = 0.625 and K = 0.61, respectively).

### Evaluation of patients’ current health status by physicians and patients

Current health status (as measured by EQ-VAS) was rated higher by both patients and physicians in the PCC setting compared to the MDC setting (*p* < 0.01, Table 2); however, high concordance was found between patients’ ratings of EQ-VAS and physicians’ ratings of the same assessment tool in both treatment settings (*r* = 0.72, *p* < 0.01 for MDCs and *r* = 0.83, *p* < 0.01 for PPCs).

Multivariate linear regression analysis using the stepwise-selection approach was used to determine the factors that contributed the most to the difference between physician-estimated and patient-reported EQ-VAS. The final model included six independent predictor variables that significantly explained 32% of the observed variance in difference between patients’ and physician-estimated current health status (Table 3). The analysis showed that the mean relative score difference decreased with older age, shorter disease duration, lower HbA1c level, less diabetes-related complications, better patient-reported familiarity, and worse physician-estimated diabetes-specific QoL. Diabetes duration was the most significant contributing factor for the mean relative score difference, explaining 16% of the variance (F(1,105) = 19.14, *p* < 0.01). No statistically significant interactions were found between the independent variables included in the model (Table 3).

**Table 3:** Multivariate linear regression analysis using the stepwise-selection to determine the factors that contributed the most to the difference between physician-estimated and patient-reported EQ-VAS (*N* = 105)^a^

Predictors	B	Standard error	Partial R^2^	*P* value
Diabetes duration	0.55	0.16	0.1567	*P* < 0.01
HbA1C	1.87	0.87	0.0564	*P* < 0.01
Number of diabetes complications	1.26	0.44	0.0330	*P* < 0.01
Age	−0.23	0.13	0.0227	NS
Patient-perceived physician familiarity	−1.34	0.56	0.0160	*P* < 0.01
Physician-estimated diabetes-specific QoL	−4.25	1.91	0.0343	*P* < 0.01

*R* = 0.5649, R^2^ = 0.3191, Adjusted R^2^ = 27.74%

*NS* not significant

^a^missing values were not included in the regression analysis

### Evaluation of patients’ diabetes-specific QoL by physicians and patients

Both patients and physicians assessed the patients’ diabetes-specific QoL in the PCC setting as better than that of patients in the MDC setting (*p* < 0.01; Table 2). Concordance between patients’ and physician-estimated diabetes-specific QoL was significant in both treatment settings, but was higher in MDCs (*r* = 0.67, *p* < 0.01 for MDCs vs. *r* = 0.35, *p* < 0.01 for PCCs). Of note is that primary care physicians tended to assess patients’ diabetes-specific QoL as better than did the patients themselves, whereas diabetes specialists tended to assess it as worse than the patients.

Multivariate linear regression analysis using the stepwise-selection approach was used to determine the factors that contributed the most to the difference between patients’ and physician-estimated diabetes-specific QoL, as measured by DQoL–BCI score. The final model included five independent predictor variables that significantly explained 22% of the observed variance in difference between patients’ and physicians-estimated ratings of diabetes-specific QoL (Table 4). According to this analysis, the mean relative score difference decreased with less diabetes-related complications, higher patient-reported EQ-VAS, better physician-estimated familiarity, and worse physician-estimated PDDT. Physician-estimated diabetes-specific QoL was the most significant contributing factor for mean relative score difference - explaining 11% of the variance (F(1,105) = 12.59, *p* < 0.01). No statistically significant interactions were found between the independent variables included in the model (Table 4).

**Table 4:** Multivariate linear regression analysis using the stepwise-selection to determine the factors that contributed the most to the difference between physician-estimated and patient-reported diabetes-specific QoL (*N* = 105)^a^

Predictors	B	Standard error	Partial R^2^	*P* value
Number of diabetes complications	2.63	0.95	0.0355	*P* < 0.01
Physician-estimated QoL	12.18-	4.22	0.1060	*P* < 0.01
Physician-perceived familiarity with patients	3.19-	1.61	0.0262	*P *< 0.01
Patient-reported current health status	0.37-	0.19	0.0187	NS
Physician-estimated difficulties in diabetes treatment	6.72	3.57	0.0281	NS

*R* = 0.4631, R^2^ = 0.2145, Adjusted R^2^ = 21.45%

*NS* not significant

^a^missing values were not included in the regression analysis

### Perception of difficulty with diabetes treatment

Both patients and physicians in the MDC setting perceived the characteristics of diabetes treatment as more difficult than that of patients and physicians in the PCC setting (*p* < 0.01, Table 2). Physicians, as a whole, perceived diabetes treatment as more difficult than the patients themselves, and a strong correlation was found between patient and physician PDDT in each of the clinic settings (*r* = 0.49, *p* < 0.01 for MDCs and *r* = 0.52, *p* < 0.01 for PPCs).

### Physician-patient relationship

Overall, physicians estimated their familiarity with patients as higher than the patients themselves perceived it to be. Perceived physician familiarity among patients and physicians was higher in PCCs compared to MDCs (*p* < 0.01, Table 5). Moderate concordance between patient and physician-estimated familiarity was found for each treatment setting (*r* = 0.33, *p* < 0.01 for MDCs and *r* = 0.44, *p* < 0.01 for PCCs). This difference was significant (p < 0.01) in both care settings. In the multivariate regression model, familiarity did not explain physicians’ ability to assess health-related QoL or Diabetes-specific QoL.

**Table 5:** Patient relationship with the treating physician and clinic’s medical staff

	Total population *N* = 136	Diabetes clinic (MDC) *N* = 66	Primary care clinic (PCC) *N* = 70	*P* value
Number of patients with high levels of satisfaction with relationship with medical staff N(%)	91 (66.9)	33 (50.0)	58 (82.9)	< 0.01
Number of patients whose physician is the primary source of information regarding diabetes N(%)	103 (75.7)	45 (68.2)	58 (82.9)	< 0.01
Patient’s perception of physician’s familiarity with their condition Mean (SD)	7.65 (2.03)	6.82 (1.99)	9.05 (1.12)	< 0.01
Physician’s perception of physician’s familiarity with patient condition Mean (SD)	8.03 (1.39)	7.70 (1.46)	8.59 (1.07)	< 0.01

Categorical variables are presented as n (%) and continuous variables are presented as mean (standard deviation)

A significantly higher proportion of patients in PCCs, compared to MDCs, indicated that their physician is the primary source of information regarding diabetes (82.9% vs. 68.2%, *p* < 0.01, Table 5). Furthermore, a greater proportion of patients in PCCs than in MDCs indicated high satisfaction levels with their relationship with the clinic’s medical staff (82.9% vs. 50.0%, *p* < 0.01; Table 5).

## Discussion

It is generally agreed that patients are the most valid source of information about their own QoL [22], therefore we considered the patient-reported ratings as ‘gold standard’ to which the physicians’ assessments should be compared. In this study we showed that while primary care physicians were better at assessing their patients’overall wellbeing, diabetes-specialists were better at assessing patients’ diabetes-specific QoL. This was despite the fact that primary care physicians were more familiar with their patients than diabetes-specialists. This may well reflect the different training of these physician groups or familiarity of these physicians with different aspects of their patients’ lives. Also, the disease-specific assessment offers greater sensitivity than the generic one, which corresponds with the fact that diabetes specialists are focused on diabetes whereas primary-care physicians have a more holistic point of view.

Interestingly, the physicians felt that they were more familiar with the patients’ life situation than in fact was the case, as assessed by the patient. When assessing the patients’ current health status, the physicians were not in concordance with their patients, and perceived their patients’ QoL to be worse than their patients’ own assessment. In clinical practice this may mean that physicians may not recommend treatment that involves injecting medication (such as GLP1 agonists or insulin, prescribed mainly by specialists) for fear of worsening their patients’ QoL. This finding should encourage all physicians to specifically ask about their patients’ home and work environment and their current perception of their health status. Objectively, patients treated in MDCs were sicker: they had longer duration of diabetes, with more comorbidities and complications. In addition, almost half the patients treated in MDCs compared to less than 10 % of patients in PCCs, were treated with insulin. The patient purchase of prescribed medication was lower in the MDC than in the PCC. This may well be a reflection of the poorer diabetes-specific QoL of these patients, and may explain the higher LDL cholesterol levels in the MDC group. Diabetes duration was the most significant contributing factor for the mean relative score difference explaining 16% of the variance between patient and physician-estimated current health status using the EQ-VAS. This suggests that the longer the duration of diabetes, the harder it is to accurately assess someone else’s QoL. When the physicians tried to assess the diabetes-specific quality of life, the primary care physicians tended to underestimate the effect of diabetes on the QoL whereas the diabetes specialists tended to overestimate this effect. This finding may be attributed to the fact that sicker patients were treated in the MDCs.

The finding that physicians have greater difficulty assessing QoL of their sicker patients is not surprising in light of previous studies that have shown similar results. Parents of children with chronic health conditions (such as diabetes) typically rate their child’s health-related QoL as worse than the children themselves [23–26]. A somewhat similar pattern was observed among family members of patients with dementia, where caregivers’ ratings were, on average, lower than the patients’ ratings [27, 28]. Another notable pattern was described by Eiser and Morse in their systematic literature review, showing that concordance was better between proxy and chronically ill patients compared with proxy and healthy individuals, having greater agreement for observable functioning (e.g. physical domains), and less for non-observable functioning (e.g. social or emotional domains) [29].

Physicians’ perception and understanding of their patients’ QoL and general well-being are especially important, because physicians provide patients with treatment recommendations. Studies that compared patients’ and physicians’ ratings of health-related QoL in various diseases, showed poor concordance and lack of directionality, as in some studies the physicians rated the patients’ health-related QoL as better than the patients themselves and in some, worse [30, 31]. The agreement appeared to be lower for the subjective domains (emotion, cognition, and pain) than for the objective domains (sensation, mobility, and self-care). To the best of our knowledge, the present study seems to be the first one to deal with diabetes treatment in community clinics, where the physician is often the only source of his patients’ diabetes care.

The strengths of our study were the assessment of QoL in patients with diabetes in different settings by primary care physicians as well as specialists. This is particularly important in light of the fact that most patients with diabetes are treated in primary care. We used established questionnaires that have been fully validated in Hebrew. Since each patient filled out the questionnaire with a trained interviewer and as there were no more than 6 patients interviewed per physician, there were no repeated measures. The study is limited by the fact that not all physicians completed an equal number of assessments and sometimes the physician did not fill in his questionnaire on the same day of the patient’s visit, which may have caused a recall bias.

## Conclusions

Overall, both primary care physicians and diabetes specialists, made reasonable assessments of their patients QoL. The diabetes-specialists assessed diabetes-related QoL better than primary care physicians, while the primary care physician assessed their patients’ overall wellbeing better than diabetes specialists. However physicians tended to overestimate the negative impact of their patients’ QoL. These findings should encourage all physicians to specifically ask about their patients’ home and work environments and their current perception of their health status.

## Abbreviations

DQOL–BCI: Diabetes QoL Brief Clinical Inventory questionnaire
EQ-5D-3 L: European Quality of Life 5-Dimensions 3-Levels
EQ-VAS: European quality visual analogue scale
HbA1c: glycated hemoglobin A1c
LDL-cholesterol: low-density lipoprotein cholesterol
MDC: Multi-disciplinary diabetes-specialty clinics
PCC: Primary care clinics
PDDT: Perception of the difficulties associated with diabetes treatment characteristics
QoL: Quality of life
